# Precision and linearity targets for validation of an IFNγ ELISPOT, cytokine flow cytometry, and tetramer assay using CMV peptides

**DOI:** 10.1186/1471-2172-9-9

**Published:** 2008-03-17

**Authors:** Holden T Maecker, Jeffrey Hassler, Janice K Payne, Amanda Summers, Karrie Comatas, Manar Ghanayem, Michael A Morse, Timothy M Clay, Herbert K Lyerly, Sonny Bhatia, Smita A Ghanekar, Vernon C Maino, Corazon delaRosa, Mary L Disis

**Affiliations:** 1 BD Biosciences, San Jose, CA, USA; 2 Cancer Research and Biostatistics, Seattle, WA, USA; 3 Beckman-Coulter, San Diego, CA, USA; 4 Departments of Surgery, Medicine, Pathology, and Immunology, and Duke Comprehensive Cancer Center, Duke University Medical Center, Durham, NC, USA; 5 Tumor Vaccine Group, Division of Oncology, University of Washington, Seattle, WA, USA

## Abstract

**Background:**

Single-cell assays of immune function are increasingly used to monitor T cell responses in immunotherapy clinical trials. Standardization and validation of such assays are therefore important to interpretation of the clinical trial data. Here we assess the levels of intra-assay, inter-assay, and inter-operator precision, as well as linearity, of CD8+ T cell IFNγ-based ELISPOT and cytokine flow cytometry (CFC), as well as tetramer assays.

**Results:**

Precision was measured in cryopreserved PBMC with a low, medium, or high response level to a CMV pp65 peptide or peptide mixture. Intra-assay precision was assessed using 6 replicates per assay; inter-assay precision was assessed by performing 8 assays on different days; and inter-operator precision was assessed using 3 different operators working on the same day. Percent CV values ranged from 4% to 133% depending upon the assay and response level. Linearity was measured by diluting PBMC from a high responder into PBMC from a non-responder, and yielded R^2 ^values from 0.85 to 0.99 depending upon the assay and antigen.

**Conclusion:**

These data provide target values for precision and linearity of single-cell assays for those wishing to validate these assays in their own laboratories. They also allow for comparison of the precision and linearity of ELISPOT, CFC, and tetramer across a range of response levels. There was a trend toward tetramer assays showing the highest precision, followed closely by CFC, and then ELISPOT; while all three assays had similar linearity. These findings are contingent upon the use of optimized protocols for each assay.

## Background

Validation of immunological assays can take a number of forms, and is required for compliance with Good Laboratory Practice (GLP), or for submission of data to licensing agencies. Two basic components of assay validation are the demonstration that an assay performs with adequate reproducibility for the intended purpose, and that the assay readout is linear over a useful range of data [1].

Specific guidelines exist for validation of traditional immunoassays such as ELISA, and the expected levels of precision and linearity of these assays are well-known [2,3]. Less well-characterized are cellular immunity assays, of which the single-cell assays like tetramer staining [4], cytokine flow cytometry (CFC) [5,6], and ELISPOT [7] are among the most popular. Some data has been published regarding the precision of individual assays [8-10], and there is very limited data on the linearity of CFC [11]. However, precision and linearity have not been compared across assays, and expected levels of precision and linearity of these assays have not been determined in a side-by-side fashion.

Precision and linearity are important aspects of cellular immunity assays, since (a) cellular assays are inherently more complex, and thus less reproducible, than traditional immunoassays; and (b) cellular immunity assays are frequently used to detect rare antigen-specific T cell populations, which may be present at or near the assay detection limit. It is thus crucial to demonstrate that an assay is reproducible enough to generate reliable data in the response range expected for, say, a vaccine clinical trial, and that linearity is adequate to quantitatively compare results between treatment groups or between trials.

Fortunately, we now know that at least some vaccines to HIV and cancer, for example, can generate readily detectable T cell responses by assays such as tetramer, CFC, and ELISPOT [12,13]. Still, there is wide variability in the performance of such assays between labs [14]. Compliance with GLP thus requires that a given lab demonstrate its proficiency for a given assay, preferably with reference to an accepted standard.

Here we compare results from optimized protocols for tetramer staining, CFC, and ELISPOT, performed on shared cryopreserved PBMC specimens, with expert laboratories performing the individual assays. From this data, we derive target values for those who wish to determine precision and linearity of these assays in their own laboratory, and we also facilitate comparison of the three assays with regard to their relative precision and linearity.

## Results

### Study design and response levels of donors

In order to allow meaningful comparisons between assays, this study was performed using a format previously published [15], in which three laboratories, each expert at an individual assay, performed their assay of expertise in parallel on the same cryopreserved PBMC. PBMC from healthy CMV seropositive donors were chosen to represent a high, medium, and low responder to CMV pp65_495–503 _peptide [16] and/or a CMV pp65 peptide mix [17]. Actual mean responses across all the assays as obtained in this study are shown in Table 1. Assays were performed with six replicates in order to determine intra-assay precision. They were repeated on eight different days in order to determine inter-assay precision. Three operators performed assays in parallel on a single day in order to determine inter-operator precision. And triplicate samples from the high responder were serially diluted into non-responsive PBMC in order to determine linearity. Results were then collated across the three laboratories.

**Table 1:** Mean response levels of the three CMV-responsive donors

	% CD3^+^CD8^+ ^cells	ELISPOT (peptide mix)	CFC (peptide mix)	CFC (pp65_495–503_)	Tetramer (pp65_495–503_)	ELISPOT (pp65_495–503_)
Donor 41 (low)	20.3%	270 SFC^1 ^[0.53%]	nd^2^	0.06%^2^	0.06%^2^	8 SFC^1 ^[0.02%]
Donor 68 (med)	12.1%	285 SFC^1 ^[0.94%]	0.28%^2^	0.28%^2^	0.25%^2^	31 SFC^1 ^[0.10%]
Donor 43 (high)	26.3%	95 SFC^1 ^[0.14%]	0.87%^2^	0.93%^2^	1.27%^2^	28 SFC^1 ^[0.04%]

^1 ^ELISPOT results reported as SFC per 2.5 × 10^5 ^PBMC. Numbers in brackets indicate the values when re-calculated as % of CD8^+ ^T cells. Note that ELISPOT responses with peptide mix did not match the other assays in terms of low, medium, and high responders.

^2 ^CFC and tetramer results reported as % of CD8^+ ^T cells. nd = CFC was not done on this donor due to predicted very low response (0.02%) seen in pre-screening.

### Precision of individual assays

**Figure 1:**
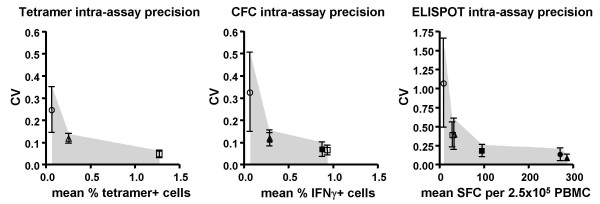
**Intra-assay CVs**. The mean CV for six replicates was plotted for samples from three donors and two different antigen stimulations in tetramer, CFC, and ELISPOT assays. Circles represent donor 41; triangles, donor 68; and squares, donor 43. Open symbols represent CMV pp65_495–503 _responses; closed symbols represent CMV pp65 peptide mix responses (CFC and ELISPOT assays only). Note that certain responses were very similar, so some symbols overlap. Error bars represent the SD of 10 times that the six replicates were repeated. The gray zones indicate the area within which a laboratory doing validation could expect their data to lie.

When the coefficient of variation (CV) of six parallel replicates was plotted against the mean response level, a characteristic non-linear relationship was observed for all three assays (Figure 1), whereby the CV rose dramatically as the mean approached zero. Since the six replicates were performed on eight separate days, a standard deviation (SD) could be determined for the intra-assay CV. Taking the mean+SD of the intra-assay CV at any given response level allows one to determine an "acceptable zone" wherein a laboratory validating these assays might expect their data to lie (gray zone in Figure 1). For example, at a response level of 0.25%, one would expect a CV no higher than about 0.15 (15%) for either tetramer or CFC, based on these data. Similarly, for a response level of about 30 SFC in ELISPOT, one would expect a CV no higher than about 0.60 (60%).

**Figure 2:**
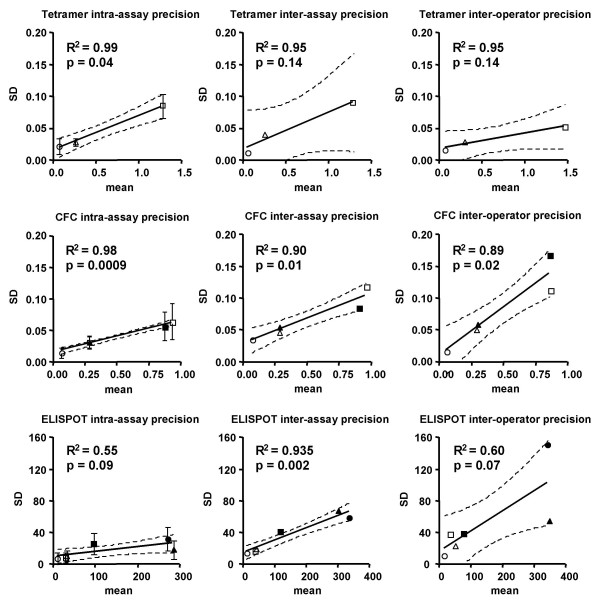
**SD of each assay**. The mean response for each sample (as per Figure 1) was plotted versus the SD of six replicates (intra-assay precision), eight assays on different days (inter-assay precision), or three operators on the same day (inter-operator precision). Circles represent donor 41; triangles, donor 68; and squares, donor 43. Open symbols represent CMV pp65_495–503 _responses; closed symbols represent CMV pp65 peptide mix responses (CFC and ELISPOT assays only). Error bars in the intra-assay graphs represent the SD of 10 times that the six replicates were repeated. Lines represent linear regression of the combined data (both antigens, where used), with 95% confidence intervals of the regression shown with dotted lines.

Because antigen-specific assays are often used to analyze data in a range where CV is non-linear with the mean, we chose to use SD as a measure of variability in further analyses. As shown in Figure 2, the SD was relatively linear with mean for all three assays, when comparing six parallel replicates (intra-assay precision, left panels), or eight separate experiments (inter-assay precision, middle panels), or three different operators (inter-operator precision, right panels). For ELISPOT, the SD was significantly lower for intra-assay precision compared to inter-assay (p = 0.03) and inter-operator (p = 0.03) precision. There was a similar trend for CFC, though the comparisons did not reach statistical significance (p = 0.06). Tetramer assays had low SD in all three situations, with no significant differences among intra-assay, inter-assay, and inter-operator precision (0.25 < p < 0.75).

### Comparative precision of the three assays

**Figure 3:**
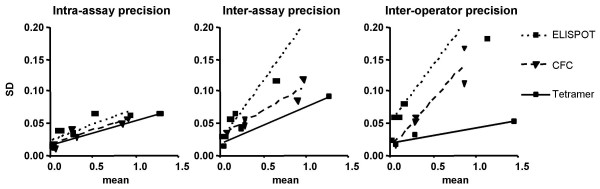
**Comparison of precision across assays**. ELISPOT means and SD for each sample were converted to percent of CD8^+ ^T cells using the formula SFC/2.5 × 10^5^/(CD8 percent of PBMC for that donor)×100. This allowed SD to be compared on the same scale. Note that the regression line for ELISPOT (dotted) is consistently higher than the regression line for CFC (dashed) or tetramer (solid). Due to complexity, data points for different donors and different antigens are not distinguished in this Figure; please refer to Figure 2 for relative responses of individual donors and antigens.

In Figure 2, it is difficult to compare the precision across all three assays, because the readout for ELISPOT (SFC per 2.5 × 10^5 ^PBMC) is different than the readout for CFC and tetramer staining (percent of CD8^+ ^T cells). In order to compare all three assays on the same scale, the ELISPOT values were converted to a percent scale (since the percentage of CD8^+ ^T cells in each PBMC sample was known). The result (Figure 3) allows visual comparison of the precision between assays. For all three conditions (intra-assay, inter-assay, and inter-operator precision), ELISPOT tended to have higher SD than CFC or tetramer. This trend was greatest for inter-assay and inter-operator studies.

### Linearity

**Figure 4:**
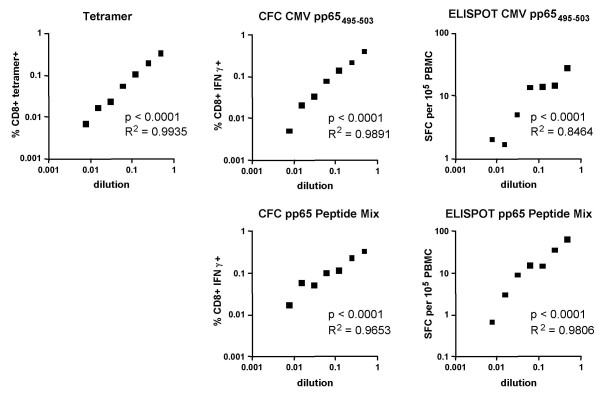
**Linearity of assays**. Triplicate samples of PBMC from a HLA-A2+ high-responding donor (#43) were serially diluted into PBMC from a known HLA-A2+ non-responder. The same non-responding donor was used for all assays. Unstimulated background was subtracted for each dilution point in CFC and ELISPOT assays. This background was uniformly low (< 0.08% for CFC and < 15 SFC per 2.5 × 10^5 ^PBMC for ELISPOT) despite the used of allogeneic PBMC for the dilution. Note that the pp65_495–503 _peptide response of this donor (#43) was much lower in ELISPOT compared to the other two assays.

Figure 4 shows the results of linearity studies, in which PBMC from a CMV-responder (donor 43) were diluted into PBMC from a known CMV non-responder. This design was chosen to mimic physiological conditions of donors with few responsive cells in the context of many non-responsive cells. It was also possible to take this approach, since allogeneic responses were not detected using this particular donor pair, stimulation time, and cytokine readout (i.e., backgrounds were as low as those seen in precision experiments with the CMV-positive donor alone). All three assays showed highly significant linearity (p < 0.0001). For the pp65_495–503 _peptide system, CFC and tetramer showed almost identical R^2 ^values (R^2 ^= 0.99), while ELISPOT was lower (R^2 ^= 0.85). This could relate to the relatively low pp65_495–503 _response of the chosen donor in ELISPOT versus the other two assays. For the pp65 peptide mix system, where the ELISPOT response of this donor was higher, ELISPOT and CFC showed similar R^2 ^values (0.97–0.98).

## Discussion

A number of studies have been published comparing ELISPOT, CFC, and tetramer staining [15,18-27]. However, while these studies have addressed issues such as relative sensitivity of the assays, direct comparisons of precision and linearity have not been done. The best previous assessments of inter-laboratory precision came from standardization studies carried out for ELISPOT [14,28] or CFC [10]. The present study was designed to compare these assays with regard to precision and linearity, and in so doing, to provide target values for laboratories wishing to validate their own protocols for any of these assays. It should be noted that these targets may be specific to IFNγ and to the antigen systems used (CMV pp65_495–503 _and pp65 peptde mix), and can not necessarily be generalized to other cytokines and antigens.

In comparing assays, the choice of protocol is crucial; one wishes to compare fully-optimized versions of each assay. As such, we chose individual labs recognized as experts in each type of assay (tetramer, CFC, and ELISPOT) and allowed them each to use their own optimized protocol. In the case of ELISPOT, this protocol did not use costimulatory antibodies, while in CFC it did. Validation data in the lab doing ELISPOT showed that inclusion of costimulatory antibodies was not warranted based on increased backgrounds (data not shown); while similar data in the CFC lab supported the use of costimulation. The net result, that the assays might not give quantitatively equivalent results, was accepted in favour of the idea that each assay was optimally configured for its own best performance.

Often, CV has been used as a measure of precision that is independent of the units in which data is expressed (e.g., SFC or percent positive cells) [29]. However, the stability of the CV deteriorates at very low means (Figure 1), so it is difficult to use it as a robust measurement of precision for low-level responses. Since antigen-specific assays are often used to assess very low response values, we chose instead to focus on SD, which we found to be highly linear with the response mean (Figure 2).

In general, there was a trend toward higher precision in intra-assay measurements compared to inter-assay or inter-operator measurements, in agreement with a previous study on CFC [11]. Since the inter-assay and inter-operator precision studies were not repeated multiple times, we could not derive statistical tolerances around the SD values in Figure 2. However, a laboratory wishing to validate these assays could use this figure as a guide, comparing their own internal validation data to the figure for any given response level.

In order to compare precision across all three assays, ELISPOT values were converted to the same scale as CFC and tetramer staining (Figure 3), and the SD was plotted versus mean. Deriving a regression line through this data for each assay, we observed that ELISPOT SD tended to be higher, for any given response level, than CFC or tetramer SD. While we did not validate this trend statistically, it is concordant with previous studies that reported relatively low inter-laboratory precision for ELISPOT [14] and relatively high inter-laboratory precision for CFC [10], at least when data were analyzed centrally.

Why would ELISPOT assays have poorer precision in this study? One relevant factor could be the number of cells collected per data point, since assay precision is dependent upon counting statistics. The ELISPOT assay was optimized such that 2 × 10^5 ^PBMC per well gave the highest counting efficiency. CFC assays collected 40,000 CD3^+^CD8^+ ^cells per sample, and tetramer assays collected 30,000 CD8^+ ^T cells. Assuming CD3^+^CD8^+ ^cells to be about 15% of PBMC, this means that roughly 30,000 CD3^+^CD8^+ ^cells were present in each ELISPOT well. While the difference relative to CFC (30,000 versus 40,000) could explain some difference in precision, it is unlikely to be a major factor, at least at the higher response levels tested.

Another factor in ELISPOT assays is the fact that precision is dependent upon cell counting and pipetting. All calculation is based upon the assumption that 2 × 10^5 ^PBMC were in fact plated in every well. If the original PBMC count were in error, this would introduce a systematic bias in that assay's results that would potentially affect inter-assay or inter-operator reproducibility. And if pipetting were not precise, intra-assay reproducibility would be affected as well. In contrast, tetramer and CFC assays have a percentage readout that is less dependent upon the true number of cells plated per sample.

The ELISPOT assay is also unique in that it is a monolayer assay, and cell stimulation could be limited by the ability of T cells and APC to interact when they are spread out over a filter-bottom well. This could affect the reproducibility of stimulation, and could also explain undercounting of responsive T cells in ELISPOT relative to CFC. Lack of costimulatory antibodies in the ELISPOT assay, which were used in CFC, could also cause undercounting. Fnally, undercounting could result from coincident spots or other imprecisions in the computer-aided ELISPOT counting system. We saw such undercounting to a variable degree in this study, when ELISPOT results were converted to the same scale as CFC and tetramer results (see Table 1). ELISPOT has been shown in other studies to undercount positive events by 4- to 10-fold relative to CFC or tetramer staining [23,24], even in the same CMV system used for the current study [15]. Thus, the ELISPOT regression line for SD versus mean (Figure 3) could be expected to shift leftward relative to CFC and tetramer, if SD for a given sample were actually equal between the three assays.

For all the above reasons, it is not surprising that ELISPOT precision was not as high as CFC and tetramer. In fact, common practice in the field takes this into account, in that ELISPOT assays are typically done with 3–6 replicates per sample, while this is rarely done in CFC and tetramer assays. It was only for purposes of fair comparison that a single well was considered as one data point in this study.

With regard to linearity, we used a dilution methodology in which responder PBMC were diluted into non-responder PBMC. There was the potential for confounding artifacts with this method, including the possibility of loss of relevant antigen-presenting cells with serial dilution, and the possibility of allogeneic responses. Fortunately, these were not observed as significant factors. Linearity of CFC and ELISPOT were similar to tetramer, except in the instance where ELISPOT response was disproportionately low compared to the CFC or tetramer response. This suggests that antigen-presenting cell frequency was not a major limiting factor. In terms of allogeneic responses, unstimulated CFC background at each dilution was 0–0.07% of CD8+ T cells, which was not different from background in the assays that did not contain allogeneic cells. For ELISPOT, the average background in the linearity studies was 4.7 SFC per 2.5 × 10^5 ^PBMC, compared to 5.0 SFC when donor 43 (high responder) PBMC were tested alone.

In terms of assay comparison, it is worth noting that the ELISPOT assay does not easily allow for phenotyping of responsive cells as CD4^+ ^or CD8^+^, etc. In our study, we fortuitously chose donors whose pp65 peptide mix responses were almost entirely CD8-restricted (pp65_495–503 _peptide responses should be CD8-restricted by nature). We thus focused on CD8+ T cell responses for all CFC assays. However, when using either CMV pp65_495–503 _or peptide mix stimulation, we observed that the rank order of donors was different for ELISPOT compared to the other two assays. CD4 responses in CFC were 0.2–0.4% for all donors (data not shown), so this was not sufficient to account for the difference. One outcome of this anomaly is that the pp65_495–503 _linearity data for ELISPOT was generated with a donor whose ELISPOT response was many fold lower than that donor's CFC or tetramer response. We thus focused more attention on the pp65 peptide mix ELISPOT response, which was at least somewhat higher, and which showed comparable linearity to CFC and tetramer.

Tetramer staining is by far the simplest of the three assays, requiring only phenotypic staining of PBMC. It is therefore not unexpected that it would have the highest precision, as the complexity of activation and sample processing should negatively impact precision of CFC and ELISPOT. However, it is interesting that CFC precision and linearity were not much different from that of tetramer staining. This is consistent with the notion that precision of CFC assays is largely related to gating and analysis, rather than activation or processing variables [10].

Although our focus in this paper is on precision and linearity, a limit of detection for each assay can also be determined from our data. This limit could be defined, for example, as 2 SD above the mean of replicate negative control samples. Such a limit is of course dependent upon the number of events collected, as previously described for CFC [30], as well as the negative control background, which varies between donors. In our study, we calculated an average lower limit of detection of 0.08% for CD8+ T cell IFNγ production in CFC, and 64 SFC per 2.5 × 10^5 ^PBMC in IFNγ ELISPOT. Note that these detection limits were the average across three donors, and were lower for those donors with low background. A similar calculation could not be done for tetramer staining, because a negative control (irrelevant tetramer) was not used.

## Conclusion

From this comparison study, we provide target values for precision and linearity of tetramer, CFC, and ELISPOT assays, using cryopreserved PBMC. We conclude that all three assays can be performed with reasonable precision and linearity. Intra-assay precision was generally lower than inter-assay or inter-operator precision. Tetramer staining tended to have the highest precision and linearity, followed closely by CFC and then ELISPOT.

## Methods

### Donors and CMV responses

Three HLA-A2^+^, CMV seropositive healthy subjects were chosen from previous work, which suggested that they represented low (~0.1% tetramer^+ ^or IFNγ^+ ^cells, or ~30 SFC per 2.5 × 10^5 ^PBMC), medium (~0.3% tetramer^+ ^or IFNγ^+ ^cells, or ~100 SFC per 2.5 × 10^5 ^PBMC), and high (~1% tetramer^+ ^or IFNγ^+ ^cells, or ~200 SFC per 2.5 × 10^5 ^PBMC) responders to CMV pp65_495–503 _peptide. Actual mean values obtained in this study are shown in Table 1.

### Collection and cryopreservation of PBMC

PBMC from leukapheresis were isolated using Ficoll gradient separation as previously described [15]. To cryopreserve PBMC, 2× freezing media was first prepared, containing 20% DMSO in RPMI (Sigma Chemical Co., St. Louis, MO) containing 12.5% human serum albumin (HSA) (Gemini Bioproducts, Woodland, CA), and cooled on ice for a minimum of 30 minutes. Ficolled PBMC at 2 × 10^7 ^viable lymphocytes/ml were resuspended in cold RPMI+12.5% HSA with no DMSO. An equal volume of chilled 2× freezing media was added to the cell suspension dropwise, while gently swirling the tube. One ml of this cell suspension was aliquoted into each cryovial (Sarstedt, Inc., Newton, NC). Once aliquoted, cryovials were placed on ice and then transferred into a freezing container (Nalgene, Rochester, NY), and stored at -80°C for 24 hours. Cryovials were then transferred into liquid nitrogen for long-term storage. After 30 days, cryovials were overnight shipped on dry ice to the recipient laboratories.

### Thawing

Cryopreserved PBMC were stored at -80°C until thawing to set up the assays. Cryopreserved cells were thawed and slowly diluted with 8 ml of warm RPMI+10% fetal bovine serum+antibiotics (cRPMI-10, all components from Sigma). The cells were centrifuged for 7 minutes at 250 × G, then resuspended as described below for each assay. Viability and recovery were checked using Trypan blue, and were > 80% and > 50%, respectively, in all samples.

### Antigens

A common source of peptide antigens was shared among laboratories for CFC and ELISPOT assays. These included CMV pp65 peptide mix (BD Biosciences, San Jose, CA; used at a final concentration of 1.7 μg/ml/peptide); and CMV pp65_495–503 _peptide (SynPep Corp., Dublin, CA; used at a final concentration of 10 μg/ml).

### Tetramer assays

Staining was done using the Multiple Antibody Single Color protocol (iMASC, Beckman Coulter Inc., Fullerton, CA) as previously described [15]. Briefly, PBMC were resuspended at 1 × 10^7 ^per mL in HBSS+0.1% bovine serum albumin+0.02% sodium azide. 100 μL of PBMC per sample were stained for 30 minutes at room temperature using CD8 FITC, HLA-A2 tetramer loaded with CMV pp65_495–503 _PE, and CD4, CD13, and CD19 PE-Cy5 (all from Beckman Coulter). Samples were washed and analyzed on a FACS Calibur flow cytometer (BD Biosciences). 30,000 CD8^+ ^T cells were collected, and results were reported as the percentage of CD8^+^, PE-Cy5-negative cells that were tetramer-positive. No correction for background staining with irrelevant tetramer was used, but the test tetramer showed undetectable staining on CMV-negative donors (not shown).

### CFC assays

Activation and processing were done as previously described [15]. Briefly, PBMC were resuspended at 1 × 10^7 ^per mL in cRPMI-10, and 200 μL were plated per well in 96-well round-bottom plates. After overnight rest at 37°C, activation reagents (stimulus+brefeldin A+costimulatory antibodies to CD28 and CD49d) were added and the cells incubated at 37°C for 6 hours. The cells were then fixed and permeabilized as per reference [15], followed by staining with IFNγ FITC/CD69 PE/CD8 PerCPCy5.5/CD3 APC (BD Biosciences) for 1 hour at room temperature in dark. Plates were washed and cells resuspended in 1% paraformaldehyde in PBS, then acquired on a FACSCalibur flow cytometer (BD Biosciences). 40,000 CD3^+^CD8^+ ^lymphocytes were collected per sample. Data were reported as the net percent of CD3^+^CD8^+^lymphocytes that were CD69^+^IFNγ^+ ^after subtracting the average response of unstimulated samples.

### ELISPOT assays

Activation and processing were done largely as previously described [15]. Briefly, Multiscreen-HA 96-well plates (Millipore, Bedford, MA) were coated with mouse anti-human IFNγ mAb, and the plates washed and blocked as per the referenced publication. PBMC were resusupended at 1 × 10^6 ^per mL, and 200 μL plated per well along with the appropriate antigen. Stimulation was for 18–24 hours at 37°C in 5% CO_2_. Plates were washed and developed as in reference [15], and the number of spots per well was determined using a KS ELISPOT Automated Reader System with KS ELISPOT 4.2 Software (Carl Zeiss, Inc., Thornwood, NY). SFC per 2.5 × 10^5 ^PBMC were reported after subtracting the average response of unstimulated samples.

### Design of precision and linearity studies

Data for each assay were acquired on a single instrument, using the same settings throughout the study. For flow cytometry-based assays, an optimized gating template and instrument settings file were used throughout the study, including inter-operator precision studies. All data for linearity, intra-assay, and inter-assay precision were performed by a single operator. For conversion of ELISPOT data to percent of CD3^+^CD8^+ ^cells, the percent of CD3^+^CD8^+ ^cells was determined from a representative subset of the CFC data, and was found to be very consistent across days and across operators. However, it varied significantly from one donor to the next (see Table 1). The ELISPOT data was converted to percent response as follows: SFC/2.5 × 10^5^/(mean CD3+CD8+ percent for that donor)×100.

### Statistical analyses

For comparison of SD among samples in intra-assay versus inter-assay or inter-operator studies, a Wilcoxon Matched Pairs test was used. Statistical analyses were carried out in GraphPad Prism software (San Diego, CA).

## Authors' contributions

HTM, JH, TMC, HKL, SAG, VCM, and MLD designed and supervised the study. JKP, AS, KC, MG, MAM, SB, and CdR obtained patient samples and collected the data. JH collated data and did statistical analyses. HTM drafted the manuscript and figures, and all authors edited and/or approved the final version.
